# Nucleolar Protein 56 Deficiency in Zebrafish Leads to Developmental Abnormalities and Anemia via *p53* and JAK2-STAT3 Signaling

**DOI:** 10.3390/biology12040538

**Published:** 2023-03-31

**Authors:** Fang Liang, Xiaochan Lu, Biyu Wu, Yexin Yang, Wei Qin

**Affiliations:** 1 Institute of Modern Aquaculture Science and Engineering, School of Life Sciences, South China Normal University, Guangzhou 510631, China; 2 Department of Otorhinolaryngology, Peking University Shenzhen Hospital, Shenzhen 518036, China; 3 Guangdong Key Laboratory of Mental Health and Cognitive Science, Key Laboratory of Brain, Cognition and Education Science, Ministry of Education of China, Institute for Brain Research and Rehabilitation, South China Normal University, Guangzhou 510631, China

**Keywords:** ribosomopathies, anemia, erythropoiesis, *nop56*, zebrafish

## Abstract

**Simple Summary:**

Ribosomopathies are a series of disorders caused by ribosomal dysfunction, usually causing tissue-specific defects such as anemia. Defects in several nucleolar proteins have been observed in human ribosomopathies. It remains to be determined whether any other ribosome proteins are involved in regulating erythropoiesis. We generated a nucleolar protein 56 (*nop56*)^−/−^ zebrafish model using the CRISPR/Cas9 system and investigated its function. A *nop56* deficiency induced severe morphological abnormalities and anemia. The erythroid lineage in definitive hematopoiesis and the maturation of erythroid cells were impaired in the *nop56* mutants. Additionally, the *p53* signaling pathway and the JAK2-STAT3 signaling pathway were abnormally activated in the mutants. The knockdown of *p53* signaling using morpholino partially rescued the malformation, and the inhibition of JAK2 partially rescued the anemic phenotype. This study suggests that *nop56* is a potential target for investigation in erythropoietic disorders, particularly those that may be associated with JAK-STAT activation.

**Abstract:**

Ribosomes are the vital molecular machine for protein translation in a cell. Defects in several nucleolar proteins have been observed in human ribosomopathies. In zebrafish, a deficiency in these ribosomal proteins often results in an anemic phenotype. It remains to be determined whether any other ribosome proteins are involved in regulating erythropoiesis. Here, we generated a nucleolar protein 56 (*nop56*)^−/−^ zebrafish model and investigated its function. A *nop56* deficiency induced severe morphological abnormalities and anemia. WISH analysis showed that the specification of the erythroid lineage in definitive hematopoiesis and the maturation of erythroid cells were impaired in the *nop56* mutants. Additionally, transcriptome analysis revealed that the *p53* signaling pathway was abnormally activated, and the injection of a *p53* morpholino partially rescued the malformation, but not the anemia. Moreover, qPCR analysis showed that the JAK2-STAT3 signaling pathway was activated in the mutants, and the inhibition of JAK2 partially rescued the anemic phenotype. This study suggests that *nop56* is a potential target for investigation in erythropoietic disorders, particularly those that may be associated with JAK-STAT activation.

## 1. Introduction

Ribosomopathies are a series of disorders caused by ribosomal dysfunction, which are often manifested as defects in ribosomal RNA modifications, ribosomal proteins, or ribosomal assembly factors [1,2]. Although the ribosome is an ubiquitous machine for mRNA translation, ribosomal dysfunction causes tissue-specific defects, most commonly erythroid failure in Diamond-Blackfan anemia and 5q-syndrome. These diseases typically present with anemia in infancy, congenital anomalies, and a risk of developing cancer. The main therapeutic methods for anemia caused by ribosomal dysfunction are the use of corticosteroids, lenalidomide, and other erythropoiesis-stimulating agents to relieve anemic symptoms, or long-term blood transfusions which pose a significant risk of infection and result in a poor prognosis [3]. Therefore, novel therapeutic approaches based on the anemia mechanism are urgently needed for ribosomopathies.

Early genetic studies of ribosomopathies in model animals such as zebrafish focused on the ribosomal biogenesis-related genes, including *rps19*, *rpl11*, *rps14*, and *rpl18*, because of their mutations in human diseases [4,5,6,7,8]. These studies demonstrated that the activation of the nucleolar surveillance pathway under ribosomal stress plays a major role in causing anemia, ultimately leading to *p53*-dependent cell cycle arrest and apoptosis [9]. Other *p53*-independent signaling pathways, including ATG5-dependent autophagy, mTOR signaling, JAK2-STAT3 signaling, and IFN signaling, are also involved in these disorders [7,10,11,12]. 

The nucleolus contains a diverse population of small nucleolar RNAs, which interact with its core proteins to constitute a mature, small nucleolar protein complex essential for ribosome biogenesis [13,14]. The nucleolar protein 56 (NOP56) is a decisive component of the box C/D small nucleolar protein complex, together with NOP58, fibrillarin, and SNU13, which promote pre-ribosomal RNA maturation and 60S ribosomal subunit assembly [15,16]. NOP56 defects impair ribosomal biogenesis, which has been associated with diverse types of human cancers [17,18]. The expansion of a hexanucleotide repeat in intron 1 of *NOP56* causes a novel type of dominant cerebellar ataxia, spinocerebellar ataxia type 36 [19,20]. Dysfunction of *nop56* in zebrafish induces a severe neurodegenerative syndrome [21]. However, there is little research to elucidate the function of *NOP56* in blood development in vivo. In this study, we generated a *nop56*^−/−^ zebrafish using the CRISPR/Cas9 system. A *Nop56* deficiency in zebrafish led to severe anemia and morphological abnormalities. The specification of the erythroid lineage in definitive hematopoiesis and the maturation of erythroid cells were also affected in the *nop56* mutants. An RNA-seq analysis revealed that the *p53* signaling pathway was abnormally activated, and inhibition of *p53* partially rescued the morphological abnormalities, but not the anemia. A qPCR analysis showed that the JAK-STAT signaling pathway was activated in the mutants. CEP-33779, a small molecular inhibitor of JAK2, rescued the anemic phenotype, which may be a candidate therapeutic drug for anemia.

## 2. Materials and Methods

### 2.1. Zebrafish Maintenance

All the zebrafish used in this study were raised in groups and maintained under standard laboratory conditions at 28.5 °C. The wildtype line used was Tubingen (TU), while the *Tg(LCR:EGFP)* transgenic line was specifically labeled erythrocytes, and the *Tg(kdrl:GRCFP)*^zn1^ transgenic line was specifically labeled vascular endothelial cells. All the zebrafish embryos and adults used in this study were chosen randomly. Embryos and adults were genotyped after analysis.

### 2.2. mRNA and gRNA Synthesis

The pT3TS-nCas9n plasmid (addgene, plasmid #46757) for zebrafish was linearized with *Xba*I. The capped zCas9 mRNA was obtained through in vitro transcription using linearized plasmids with a T3 mMESSAGE Kit (Ambion, Carlsbad, CA, USA) and purified using a RNeasy FFPE kit (Qiagen, Dusseldorf, Germany). For *nop56* mRNA synthesis, the coding sequence was amplified using cDNA and cloned into pCS2^+^ using gateway technology (Thermo Fisher Scientific, Wilmington, NC, USA). Then the mRNA was transcribed after plasmid linearization using a SP6 mMESSAGE Kit (Ambion, Carlsbad, CA, USA). All the gRNAs were transcribed in vitro using T7 RNA Polymerase (TaKaRa Bio, Shiga, Japan) and purified using a RNeasy FFPE kit (Qiagen, Dusseldorf, Germany).

### 2.3. Generation of the nop56^−/−^ Homozygous Mutants

A mixture (2 nL) containing zCas9mRNA (200 ng/µL) and gRNA (50 ng/µL) were co-injected into one cell-stage zebrafish embryos. The injected embryos were bred at 28.5 °C for observation of phenotypes and PCR amplification. These embryos were raised to adulthood as F0. The F0 adult zebrafish were outcrossed to wildtype fish and the F1 embryos were collected. For genotyping, PCR products were amplified from the genomic DNA of F1 embryos and subjected to electrophoresis with the following primers: *nop56* Fwd: 5’-GATGCACACATAATCTGTCAA-3’ and *nop56* Rev: 5’-CTAGAGAGCATTTGATTGGT-3′. The PCR products showing positive bands were further cloned and sequenced. The pairs of *nop56*^+/−^ F1 heterozygotes were incrossed and generated the *nop56*^−/−^ homozygous mutants.

### 2.4. Real-Time Quantitative PCR

The total RNA was isolated from whole embryos (30 embryos per group) at specific stages using TRIzol Reagent (Thermo Fisher Scientific, Wilmington, NC, USA) according to the manufacturer’s directions. The cDNA was made using a PrimerScript^TM^ RT reagent kit (TaKaRa Bio, Shiga, Japan) for next real-time quantitative PCR analysis. A quantitative PCR was performed on the CFX96 real-time PCR detection system (Bio-Rad, Hercules, CA, USA) using TB Green^®^ Premix Ex Taq™ (TaKaRa Bio, Shiga, Japan). Each experiment was performed in triplicate. The Primers for *nop56* are as follow: *nop56*-qPCR-F:5’-GATTGGCATGCTTCTACCTC-3’, *nop56*-qPCR-R:5’-CAGCTACCACACCTCCAGTC-3’. The other primers for the qPCR were used as previously described in [7].

### 2.5. TUNEL Staining

The embryos for TUNEL staining were fixed with 4% paraformaldehyde (PFA) overnight at 4 °C followed by three washes in PBST. Then, the embryos were treated with acetone for 5 min and digested with proteinase K (10 μg/mL, Roche, Basel, Switzerland) at room temperature for 20 min. Subsequent TUNEL staining was performed on the embryos following the manufacturer’s protocol of In Situ Cell Death Detection Kit (Roche, TMR red, Basel, Switzerland). The reaction was stopped after 1h dark treatment, and the embryos were imaged immediately.

### 2.6. o-dianisidine Staining

For *o*-dianisidine staining, the embryos were collected and fixed at room temperature for 2 h using 4% PFA. After the removal of the PFA using PBST wash, the embryos were dyed using an o-dianisidine staining solution (40% anhydrous ethanol, 0.65% hydrogen peroxide, 10 mM sodium acetate, and 0.6 mg/mL o-dianisidine [Sigma-Aldrich, Saint Louis, MO, USA]) in the dark for 30 min and followed by three washes with PBST. Then, the embryos were incubated in a bleach solution (1% potassium hydroxide, 3% hydrogen peroxide) until the pigmentation was removed. The embryos were then washed with PBST and imaged using a microscope (ZEISS, Imager.A1, Oberkochen, Germany).

### 2.7. Wright–Giemsa Staining

For Wright–Giemsa staining, the embryos were anesthetized using 0.3% tricaine and then immersed in 40% FBS (Thermo Fisher Scientific, Wilmington, NC, USA) mixed with a final concentration of 5 mM EDTA (Corning, New York, NY, USA). Red blood cells were collected from the heart using microinjection needles. The collected cells were smeared evenly on slides and air-dried rapidly. After fixing in the methanol for 5 s, the cells were soaked in Wright–Giemsa solution (BBI Life Science, Shanghai, China) for 10 min. Finally, the slides were rinsed with deionized water. Images were taken with a microscope after air-drying.

### 2.8. WISH

For the collection of embryos, embryos of different periods were fixed in 4% PFA solution overnight at 4 °C. Embryos can be stored at −20 °C for a long time after fixation and gradient. The cDNA fragment for *nop56* was cloned into pUC19 plasmid (TaKaRa Bio, Shiga, Japan) using *nop56*-probeF: 5′-TAATACGACTCACTATAGGGAGAGCTGCGAGATTTGGTGC-3′ and *nop56*-probeR: 5′-GGATCCACGTAACTGAGTGCGTTCTTTCCC-3′. Probes for other genes (*scl, lmo2*, *gata1*, *c-myb*, *hbae1.1*, *lyz*, *lcp1*) were used as previously described in [5,6]. A whole mount in situ hybridization was performed as described in [22].The stained embryos were photographed using a microscope (ZEISS, Imager.A1, Oberkochen, Germany).

### 2.9. RNA-Sequencing

The total RNA was isolated from the whole embryo (30 embryos per group) at 48 hpf using TRIzol^®^ Reagent (Thermo Fisher Scientific, Wilmington, NC, USA). Two groups of sibling and mutant samples were collected independently. The quantity of total RNA was controlled as concentrations ≥ 200 μg/μL and a purity RIN  ≥  7, 28S/18S  ≥  1.0. The mRNA enriched using Oligo (dT) beads was fragmented into fragments (200–700 nt) and reverse-transcribed into first-strand cDNA using random primers. End reparation, poly-A addition, and Illumina sequencing adapters ligation were performed on the second-strand cDNA using a QIAquick PCR Purification Kit (Qiagen, Dusseldorf, Germany). Finally, the PCR amplification of these samples was sequenced using Illumina HiSeqTM. The raw reads containing only adapter, oligo A, low quality reads (Q value ≤20), and unknown nucleotides ≥10% were removed. Then the high-quality clean reads were mapped to the zebrafish reference genome using Tophat2 (Tophat v. 2.1.1, Baltimore, MD, USA). Differential expression genes were analyzed using edgeR software (FDR < 0.05 and fold change > 2) [23] and subjected to gene ontology [24] and KEGG pathway enrichment analysis [25].

### 2.10. Inhibitor Treatments

The inhibitor of JAK2, CEP-33779, was purchased from TargetMol, Boston, MA, USA and dissolved in DMSO to a storage concentration of 10 mM. The embryos were collected at shield stage and treated with 10 μM CEP-33779 in a Holt buffer. The embryos were observed and collected for the following experiments at 2 dpf.

### 2.11. Statistics and Reproducibility

The experiments were repeated three times independently. Then, the mean and SEM were calculated. P values were calculated using a two-sided unpaired Student’s *t*-test and a value of less than 0.05 was considered to be significant.

## 3. Results

### 3.1. Loss of nop56 Using CRISPR/Cas9 Leads to Morphological Defects and Anemia in Zebrafish

**Figure 1:**
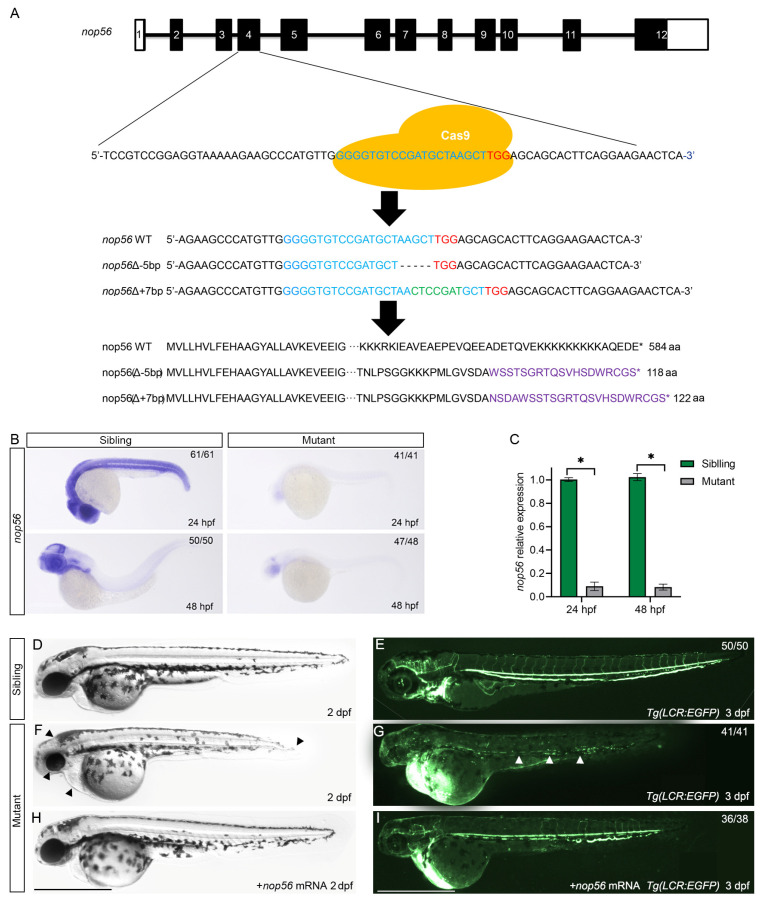
Generation of a *nop56* mutation using the CRISPR/Cas9 system. (**A**) Schematic describing the target site of *nop56*. The genomic sequences and predicted protein sequences based on the DNA sequence in the wildtype and *nop56*^−/−^ mutants are shown. (**B**) WISH of *nop56* in the siblings and mutants at 24 and 48 hpf. The results were verified independently three times. (**C**) The qRT-PCR of *nop56* transcript levels in the wildtype siblings and *nop56*^−/−^ mutants at 24 and 48 hpf. The qRT-PCR was performed with biological repeats and technical repeats in triplicate (*n* = 3, * *p* < 0.05). (**D**–**H**) In vitro-transcribed *nop56* mRNA (400 pg) partially rescued the phenotype in *nop56* mutants. A smaller head, edema in the heart, and a shorted tail extension (arrowhead) were present. (**E**–**I**) Analysis of blood cells in the *Tg(LCR:EGFP)* transgenic line. The arrowhead indicates red blood cells. All scale bars represent 1 mm.

*NOP56* encodes a nucleolar protein conserved from archaea to vertebrates, and shares 74.68%/76.13% amino acid identity between zebrafish and humans/mice, respectively (Figure S1). To explore the role of *nop56* in zebrafish development, we generated two *nop56* mutant lines by targeting the fourth exon using CRISPR/Cas9 (Figure 1A and Figure S1A). One of these mutant lines contained a 5 bp deletion that subsequently led to truncated polypeptides with only 118 amino acids. The other mutant line contained a 7 bp insertion encoding a truncated 122 amino acid protein (Figure 1A). These mutant lines were outcrossed to the wildtype and maintained as heterozygotes.

After intercrossing these *nop56*(∆-5bp) heterozygotes, morphological abnormalities were seen in the presumptive homozygous mutant embryos compared to the siblings, as indicated by pericardial edema, smaller eyes and head, and a shortened and curved body axis at 2 days post-fertilization (dpf) (Figure 1D,F). The same morphological abnormalities were also observed in the presumptive homozygous mutation embryos of the intercrossing of the *nop56*(∆+7bp) heterozygotes. To confirm the reliability of the *nop56*^−/−^ embryos, reduction of the *nop56* transcript level was assessed using whole-mount in situ hybridization (WISH) and quantitative reverse transcription-PCR (qRT-PCR) at 24 and 48 h post-fertilization (hpf) (Figure 1B,C). Next, we evaluated whether the blood cells were affected by crossing *nop56* mutants with the *Tg(LCR:EGFP)* transgenic line, and found that red blood cells tagged with EGFP were greatly reduced in the *nop56*^−/−^ embryos compared to sibling embryos at 3 dpf (Figure 1E,G). As a result, blood circulation was severely interrupted at 2 dpf (Supplementary Materials Video S1). Furthermore, both the morphological defects and anemia were mostly rescued by injection with in vitro-transcribed *nop56* mRNA in the mutant embryos (Figure 1H,I). These results indicated that the *nop56* deficiency led to the morphological defects and an anemic phenotype in the zebrafish.

### 3.2. Morphological Malformation of nop56 Deficiency Is Partially due to Increased Apoptosis

**Figure 2:**
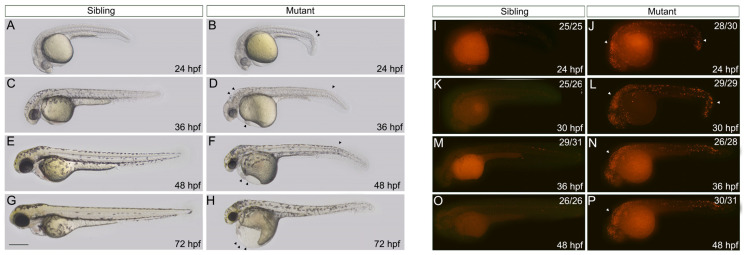
The embryos with *nop56* deficiency display morphological abnormalities due to increased apoptosis. (**A**–**H**) The phenotypes of wildtype siblings and *nop56*^−/−^ mutants. At 24 hpf (**A**,**B**), a minor curved tail (arrowhead) and slightly slower development were seen in the mutants. At 36 hpf (**C**,**D**), the ventricle and cerebellum of the mutants showed obvious bulging, and malabsorption of the yolk led to a slight swelling of the yolk sac, and pigmentation was delayed. The narrow eyes and edema in the heart were more apparent at 48 hpf (**E**,**F**). At 72 hpf (**G**,**H**), the mutants showed cardiac edema, a severely deformed trunk and yolk, and a smaller head and eyes. (**I**–**P**) The apoptosis level of the siblings and mutants at 24 hpf (**I**,**J**), 30 hpf (**K**,**L**), 36 hpf (**M**,**N**), and 48 hpf (**O**,**P**), determined using TUNEL staining. The arrowhead indicates apoptotic signals. All scale bars represent 500 μm.

To determine how the developmental abnormalities occurred in the *nop56*-deficient embryos, we observed the offspring from intercrossing heterozygous adult fish. No notable morphological differences were observed between the siblings and mutants until 21 hpf. At 24 hpf, the morphology of the *nop56*^−/−^ mutants was clearly distinguished from that of the siblings, with a minor curved tail and slightly slower development (Figure 2A,B). At 36 hpf, the ventricle and cerebellum of the mutants showed obvious bulging, and malabsorption of the yolk led to a slight swelling of the yolk sac. Additionally, narrow eyes, delayed pigmentation, and retardation of head development could be observed (Figure 2C,D). Subsequently, the mutant deformities were further aggravated at 48 hpf (Figure 2E,F). At 72 hpf, the mutants showed cardiac edema, a severely deformed trunk and yolk, and a smaller head and eyes. Moreover, the heartbeat of the mutant embryos gradually slowed until the heart stopped (Figure 2G,H). However, there was no obvious difference between the sibling and wildtype embryos in the embryonic development process.

The morphological defects caused by ribosomal stress are usually associated with increased apoptosis [6,7,26]. To determine the apoptosis level of the mutants and siblings, zebrafish embryos at 24, 30, 36, and 48 hpf were collected for TUNEL staining. A large number of apoptotic signals were detected ubiquitously in the *nop56*^−/−^ mutants at 24 hpf (Figure 2I,J). After 30 hpf, apoptotic cells were more gradually concentrated in the head and tail of the embryo (Figure 2K–P), which indicated that the tissues in these areas were undergoing apoptosis. During embryonic development, the number of apoptotic cells was constantly increasing. These results directly demonstrated that the morphological defects of the *nop56*^−/−^ mutants were partially due to severe apoptosis.

### 3.3. Maturation of Erythroid Cells and Specification of the Erythroid Lineage in Definitive Hematopoiesis Are Abnormal in nop56 Mutants

**Figure 3:**
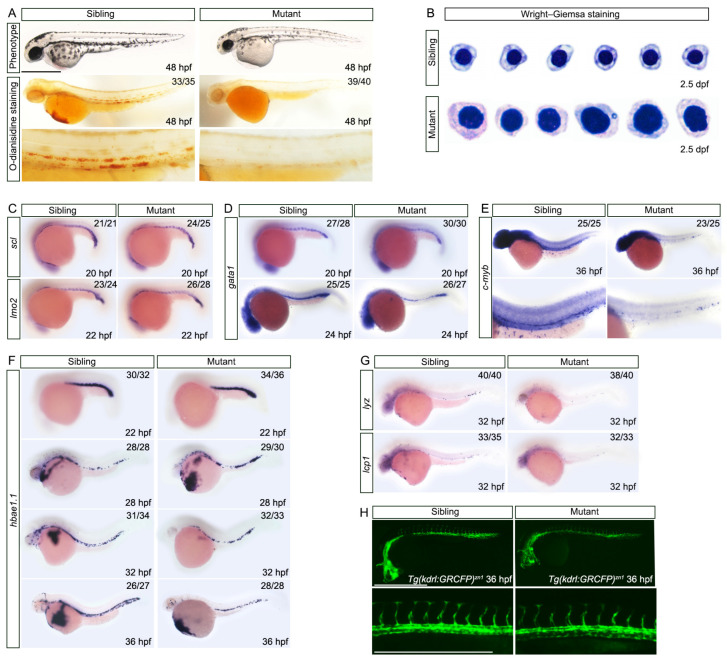
*Nop56* deficiency impairs erythroid maturation. (**A**) o-Dianisidine staining showed depletion of erythroid cells in *nop56*^−/−^ embryos compared to wildtype siblings at 48 hpf. (**B**) Wright–Giemsa staining of erythroid cells at 2.5 dpf in siblings and mutants. (**C**) WISH showed that the expression patterns of *scl* and *lmo2* were indistinguishable between siblings and mutants before 24 hpf. (**D**) WISH showed no difference in *gata1* expression in *nop56*^−/−^ embryos at 20 hpf between siblings and mutants, but a minor reduction occurred at 24 hpf in mutants. (**E**) WISH revealed that *c-myb* expression was significantly reduced from 36 hpf. (**F**) Expression of *hbae1.1* was gradually decreased from 32 to 36 hpf (*n* = 3). (**G**) WISH showed that the number of myeloid cells marked with *lyz* and *lcp1* was slightly decreased at 32 hpf. (**H**) Analysis of vasculature after crossing *nop56* mutants with the *Tg(kdrl:GRCFP)^zn1^* transgenic fish line. The vasculature development was normal in mutants. All scale bars represent 500 μm.

To explore the role of *nop56* in the development of erythroid cells, *o*-dianisidine staining was used to assess the level of anemia. We found that *o*-dianisidine staining in the mutants had almost disappeared at 48 hpf compared to the siblings (Figure 3A). To examine the cytomorphological status of the erythrocytes, we collected blood cells at 2.5 dpf for Wright–Giemsa staining. Based on the developmental stage of the erythrocytes [27], the erythroid cells of the mutants had arrested at the basophilic erythroblast stage, which should have been polychromatophilic erythroblasts after 2 dpf (Figure 3B), suggesting a delay in the maturation of the erythroid cells in the *nop56*^−/−^ mutants.

To evaluate erythropoiesis in the mutants, we performed a WISH of the hematopoietic markers at various developmental stages. In zebrafish, scl and lmo2 recruit gata1 to initiate the differentiation of primary hematopoietic erythroid cells [28,29,30]. The expression patterns of *scl*, *lmo2*, and *gata1* were indistinguishable between the siblings and mutants before 24 hpf (Figure 3C,D), indicating that the specification of the erythroid lineage from primitive hematopoiesis was unaffected. However, the expression level of *gata1* was slightly reduced at 24 hpf in the mutants, suggesting that the erythroid cells had begun to decrease (Figure 3D). c-Myb plays an important role in modulating hematopoietic stem cell migration and differentiation in definitive hematopoiesis in zebrafish [31]. In the *nop56*^−/−^ mutants, there was a dramatic decrease in *c-myb* expression at 36 hpf (Figure 3E), along with gradually decreasing globin transcripts, i.e., *hbae1.1*, from 32 to 36 hpf (Figure 3F). Additionally, there was no notable difference in the number of myeloid cells marked with *lyz* and *lcp1* at 32 hpf (Figure 3G). However, vasculature development was normal in the mutants (Figure 3H). Thus, the development of definitive hematopoiesis, especially the erythroid lineage, was impaired in the *nop56* mutants. 

### 3.4. Transcriptome Analysis of nop56 Mutants

**Figure 4:**
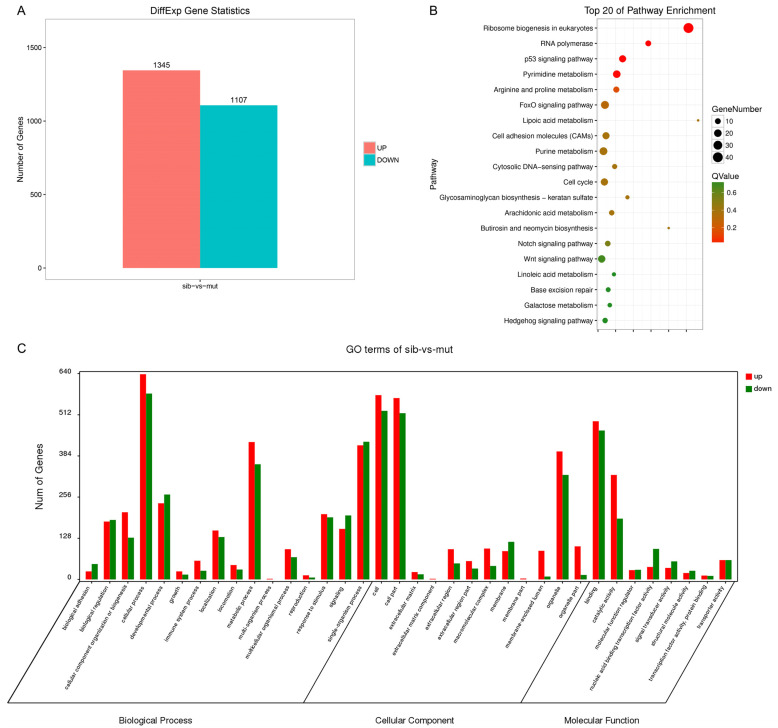
Transcriptome analysis of *nop56* mutants. (**A**) Differential expression analysis of genes between siblings and mutant groups. (**B**) Pathway enrichment analysis identified the top 20 pathways affected in mutants. (**C**) Gene Ontology analysis between siblings and mutants.

To understand the mechanism by which *nop56* deficiency induced malformations in early embryonic development, we used RNA-sequencing to compare global transcriptome variations between the siblings and mutants at 48 hpf. There were 1345 upregulated genes and 1107 downregulated genes in the mutants (fold change > 2, FDR < 0.05) (Figure 4A). KEGG pathway analysis indicated that ribosome biogenesis, the *p53* signaling pathway, the FoxO signaling pathway, and the cytosolic DNA-sensing pathway were significantly disrupted in the mutants (Figure 4B). A Gene Ontology analysis showed that the differentially expressed genes were enriched in the biological processes, including the cellular process, single organism process, and metabolic process, mainly in the cellular component containing the cell part, cell, and organelle, which affected molecular functions such as binding and catalytic activity (Figure 4C). These data suggested that *nop56* deficiency indeed led to ribosomal dysfunction and affected ribosome-related gene expression.

### 3.5. Knockdown of p53 and Inhibition of jak2 Partially Rescue Malformations and Anemia in nop56 Mutants

**Figure 5:**
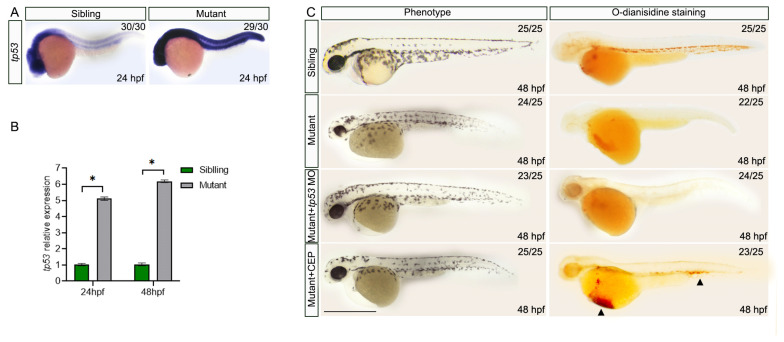
Knockdown of *p53* and inhibition of *jak2* partially rescue malformation and anemia in *nop56* mutants. (**A**) WISH analysis of *p53* in siblings and mutants. (**B**) qRT-PCR showed that *p53* expression was increased significantly in *nop56* mutants. qRT-PCR was performed with biological repeats and technical repeats in triplicate. *n* = 3, * *p* < 0.05. (**C**) Analyses of the phenotype and hemoglobin level after *p53* knockdown and CEP-33779 treatment in *nop56* mutants. Knockdown of *p53* partially rescued the morphological malformations, but not anemia, at 48 hpf. However, CEP-33779 partially rescued the anemic phenotype, but not malformations, at 48 hpf. Arrowhead indicates erythroid cells. All scale bars represent 1 mm.

Ribosomal dysfunction increases apoptosis dependent on *p53* activation [6,32,33]. Transcriptome analysis and TUNEL staining confirmed that a *nop56* deficiency caused severe apoptosis and *p53* activation in the anterior (Figure 2 and Figure 4). WISH and qRT-PCR analysis showed that *p53* was significantly increased in the *nop56* mutants (Figure 5A,B). Moreover, downstream signals of *p53*, including *mdm2*, *cdkn1a*, *casp8*, *ccng1*, and *bbc3* were significantly increased at 2 dpf (Figure S2A). To determine whether the defects in the *nop56* mutants were dependent on *p53*, we injected *p53* morpholinos at the one-cell stage of the mutants. The knockdown of *p53* partially rescued the morphological malformation, but not anemia, at 48 hpf (Figure 5C).

Previous studies have demonstrated that STAT3 competitively inhibits GATA1 binding to the 5ʹ-UTR of γ-globin in vivo, which suppresses erythroid cell maturation [34,35]. The anemic phenotype was rescued by inhibition of the JAK2-STAT3 signaling pathway in a ribosomal-deficiency model [7]. Our qRT-PCR analysis showed that the expression of *stat3* and the downstream genes of *stat3*, including *irf9*, *bcl2l1*, *mcl1a*, *mcl1b*, *pim1*, *myca*, *cycd1*, and *gfap*, was elevated to various degrees at 2 dpf (Figure S2B). The expression of genes involved in anti-apoptosis signaling, including *bcl2l1*, *mcl1a*, *mcl1b*, and *pim1*, was slightly increased, and the expression of genes involved in cell cycle progression, including *myca* and *cycd1*, was significantly elevated. However, the expression of *gfap*, which is involved in differentiation, was drastically reduced in the mutants. Moreover, a specific inhibitor of JAK2, CEP-33779 [36], partially rescued the anemic phenotype, but not the malformations (Figure 5C), suggesting that the abnormally activated JAK2-STAT3 signaling pathway affected erythroid cell maturation.

## 4. Discussion

The combined data from the *nop56*-deficient zebrafish provide a model in which *nop56* is essential for erythropoiesis in zebrafish, although no anemia cases have been reported to be related to a *nop56* mutation. A recent *nop56*^sa12582^ zebrafish line generated using the ENU method showed severe neurodegeneration characterized by the absence of the cerebellum, significantly reduced numbers of spinal cord neurons, and impaired motor functions with high levels of *p53*-dependent apoptosis in the central nervous system [21], which is consistent with our results. Additionally, we found that erythroid cell maturation was impaired in our *nop56* mutants. The *p53* signaling pathway was also significantly activated in the *nop56* mutants, because, as previous studies have shown, the pathogenesis of ribosomopathies has been linked to *p53* activation [9,10]. However, *p53* knockdown by injecting *p53* morpholinos only rescued the morphological abnormalities, but not the anemia, which is consistent with other ribosomopathy models [6,37]. These data indicate the involvement of other *p53*-independent pathways in the pathogenesis of *nop56*-induced anemia.

KEGG pathway analysis showed that the FoxO signaling pathway was significantly disrupted in the *nop56* mutants (Figure 4B). The FoxO signaling pathway is involved in the regulation of the cell cycle, apoptosis, autophagy, oxidative stress resistance, and DNA repair, and has emerged as a possible therapeutic target for aging, cancer, diabetes, and cardiovascular and neurodegenerative diseases [38]. Among the four members in mammals (FOXO1, FOXO3, FOXO4, and FOXO6), FOXO3 is critical for the terminal maturation of erythroblasts by enhancing the function of other erythroid transcription factors [39,40]. The knockdown of *foxo3b* suppresses erythroid differentiation, and the knockdown of *foxo3a* leads to neural developmental defects in zebrafish [41,42]. However, our transcriptome analysis showed that the transcript level of *foxo3b* was significantly increased, while the expression of *foxo3a* was slightly decreased (Supplementary Materials Table S1). Further experiments are needed to determine whether FoxO signaling is critical for the maturation of erythroid cells in *nop56* mutants.

The inhibition and phosphorylation of STAT3 disrupt erythrocyte differentiation and maturation [35,43]. Our previous study showed that the JAK2-STAT3 signaling pathway plays an important role in erythroid maturation in ribosomal protein-deficient zebrafish [7]. Transcriptome analysis showed that the FoxO signaling pathway-related gene *stat3* was significantly overexpressed in the *nop56* mutants (Supplementary Materials Table S1), which was confirmed using qRT-PCR (Figure S2B). The upstream genes of JAK2-STAT3, including *il6*, *il10*, and *il6st*, were expressed at a very high level in RNA-sequencing data (Supplementary Materials Table S1). IL-6 inhibits γ-globin expression, and inhibition of Stat3 phosphorylation abrogates IL-6-mediated γ-globin silencing in erythrocytes [34,44]. Our data showed that a blockade of jak2a by CEP-33779 partially rescued hemoglobin synthesis. Therefore, *nop56*-induced anemia was partially dependent on IL-6-jak2-stat3 activation. The same phenomenon have also been seen in other zebrafish models with ribosomal dysfunction [7,45]. More evidence is needed to evaluate therapies targeting IL6-jak2-stat3 signaling for a potential treatment of ribosomal deficiency-induced anemia.

## 5. Conclusions

We generated a *nop56*^−/−^ zebrafish using the CRISPR/Cas9 system. *Nop56* deficiency induced severe morphological abnormalities and anemia in zebrafish. WISH analysis showed that the specification of the erythroid lineage in definitive hematopoiesis and maturation of the erythroid cells were impaired in the *nop56* mutants. Transcriptome analysis revealed that the *p53* signaling pathway was abnormally activated, and injection of a *p53* morpholino partially rescued the malformations, but not the anemia. qPCR analysis showed that the JAK2-STAT3 signaling pathway was activated in the mutants, and inhibition of JAK2 partially rescued the anemic phenotype, which may be a potential therapeutic drug for ribosomal deficiency-induced anemia.

## Data Availability

The data that support the results of this study are available by reasonable request from the corresponding author.

## Supplementary Materials

The following supporting information can be downloaded at https://www.mdpi.com/article/10.3390/biology12040538/s1, Figure S1: The amino acid identity of *nop56* between human, mouse and zebrafish, Figure S2: qRT-PCR analysis of *p53* related genes (A), and *stat3* related genes (B) transcript levels of siblings and mutants at 48 hpf, Video S1: The blood circulation is affected in *nop56*^−/−^ mutant, Table S1: Summary of RNA-Seq data for each sample.
