# Supplementary material for: Nucleolar Protein 56 Deficiency in Zebrafish Leads to Developmental Abnormalities and Anemia via p53 and JAK2-STAT3 Signaling
Source: Biology (Basel). 2023 Mar 31;12(4):538. doi: 10.3390/biology12040538 (PMC10136036; doi:10.3390/biology12040538)
Supplement: Supplementary file 1 [file biology-12-00538-s001.zip › Supplementary Materials Figure S1 and S2.pdf]

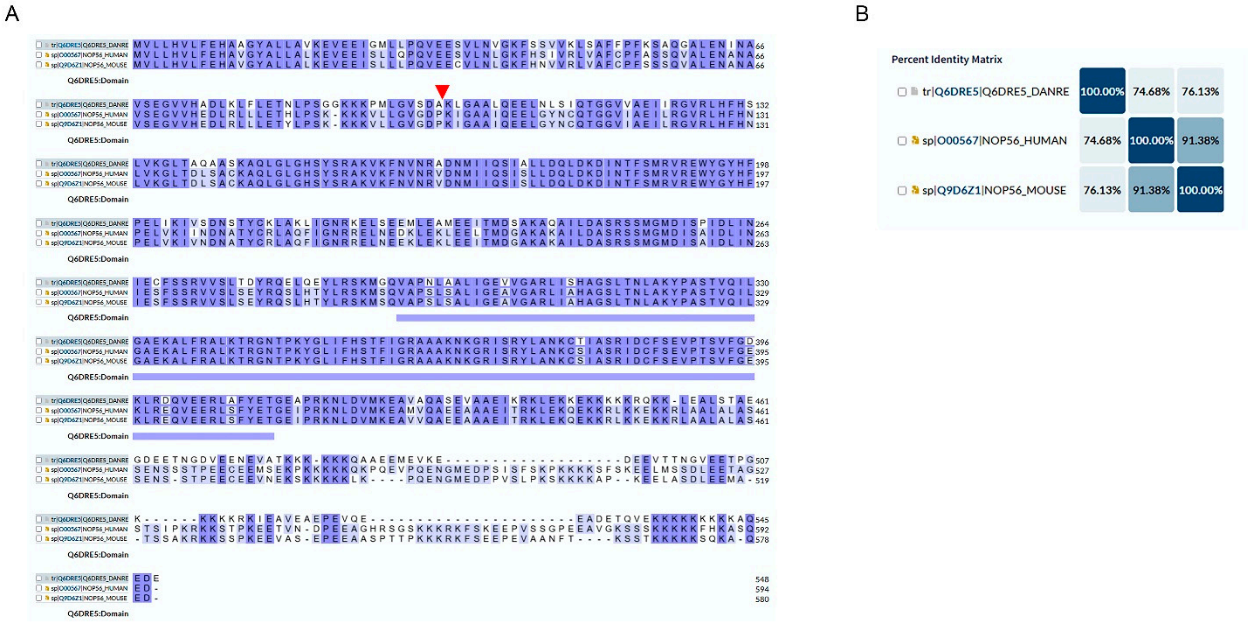

Figure S1. The amino acid identity of nop56 between human, mouse and zebrafish. (**A**), The alignment result of amino acid sequence of nop56 in human, mouse and zebrafish. Arrowhead represents the target site. (**B**), the percent identity matrix of nop56 between human, mouse and zebrafish.

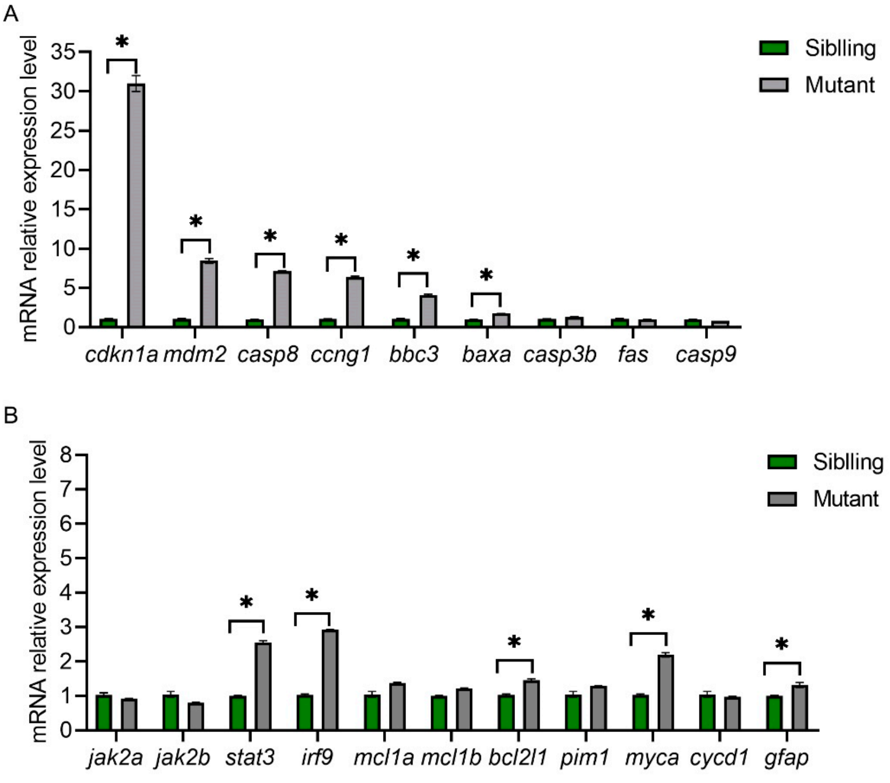

Figure S2. qRT-PCR analysis of *p53* related genes (**A**), and *stat3* related genes (**B**) transcript levels of siblings and mutants at 48 hpf. $n = 3$, $* < 0.05$.
