# Supplementary figures and images for: Nucleolar Protein 56 Deficiency in Zebrafish Leads to Developmental Abnormalities and Anemia via p53 and JAK2-STAT3 Signaling
Source: Biology (Basel). 2023 Mar 31;12(4):538. doi: 10.3390/biology12040538 (PMC10136036; doi:10.3390/biology12040538)

## Slide 1
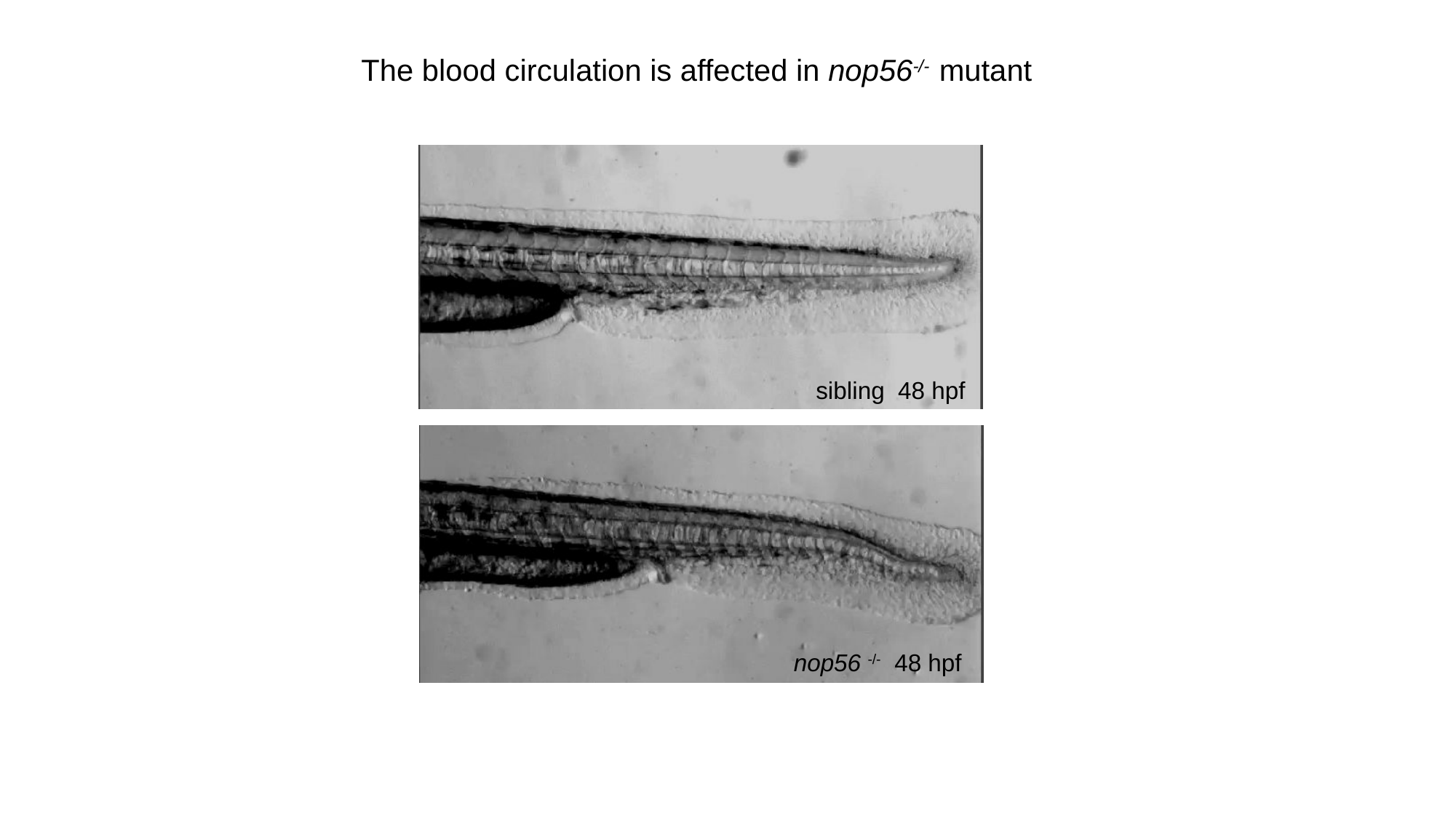

The blood circulation is affected in nop56-/- mutant
sibling 48 hpf
nop56 -/- 48 hpf

Supplement: Supplementary file 1 [file biology-12-00538-s001.zip › Supplementary Materials Video S1.pptx]
